# Dr. Clarke on Calomel in Fever

**Published:** 1810-12

**Authors:** Thos. Clark


### To the Editors of the Medical and Physical Journal.

### Dr. Clarke on Calomel in Fever.

GENTLEMEN,

*Verdun*, 18*th October*, 1810.

ON lately reading several publications on Diseases of the West-Indies, I have been extremely astonished at the quantities of calomel that have been often prescribed in the yellow fever; and still more astonished that several hundred grains of calomel have been frequently exhibited in the space of one, two, or three days, without producing any visible effect whatever. However, the same authors also acknowledge that violent salivations occasionally occurred, and reduced their patients to extreme debility, and that in these cases the only probable remedy was the removal to a more temperate climate. If they had likewise confessed that death had been frequently occasioned by the administration of such enormous quantities of calomel, I firmly believe that they would not at all have deviated from truth. But the death of these unfortunate *mercurial victims*, I presume, must have been ascribed to the violence of the yellow fever.

As I wish to avoid controversies, I shall not mention names, and *solemnly avow* that the remarks contained in this letter do not proceed from personal motives—but merely from a desire to benefit mankind by cautioning strangers against the too copious use of calomel in warm climates. Having practised both in the West and East-Indies, I hope my observations will not be deemed impertinent.

While in the West-Indies, I neither prescribed, nor knew of mercury having been administered in very large quantities in the yellow fever. However, I assert, that venereal patients, or those afflicted with hepatic dysentery, are there as easily affected by calomel as in any other climate whatever.

Since the latter end of 1795, I have been in the almost constant practice of prescribing a combination of calomel and James's powder, or some other antimonial preparation, in the incipient stages of fevers; and I do not hesitate to declare that when I have only administered 12 grains of calomel, that in 24 or 48 hours afterwards, I have frequently perceived its effects distinctly in the mouth. And in the more violent cases of fever, particularly in the East-Indies, when I have administered 18 grains of calomel in the space of three hours, I never knew an instance in which the effects of mercury on the mouth were not distinctly perceived in from

12 to 36 hours, unless when vomiting or intense purging soon took place.

Hence it must appear evident, that the result of my experience, which has been tolerably extensive, is very different from that of the West-India Practitioners alluded to. And as the constitutions of my patients, I have every reason to believe, differed very little from those of their patients, I am of opinion that there must be some reason for the very different result of our practice.

When I joined his Majesty's 65th regiment, in 1790, as Surgeon's Mate, Mr. S. S. Wilson, then Surgeon to the regiment, (now Inspector of Hospitals) informed me that if I did not either administer the medicines to the soldiers myself, or entrust their administration to some confidential person, that I should be very often deceived by them. I was soon convinced of the truth of Mr. Wilson's assertion; and afterwards during my residence in the army, I adhered strictly to the instructions he gave me; and I do not hesitate to say, that if I had not done so, I should have been often the dupe of the soldiers, *and probably might have had it in my power to relate many cases of the inefficacy of prodigious doses of calomel, or of other powerful medicines*. What tends still further to confirm me in this opinion is, that the West-India authors alluded to, generally administered the calomel in pills. Whether their administration was generally intrusted to the patients themselves, or to nurses and orderly men, does not appear. But supposing that the attendants administered the pills, even then the soldiers might very easily deceive them by concealing the pills in their mouths, &c. This I know to be a common practice amongst seamen and soldiers. Indeed, when we take into consideration the direful effects of mercury to which the soldiers must have been often eye-witnesses, it is not surprising that they should have occasionally endeavoured to deceive their attendants.

When in the East-Indies, I likewise have frequently heard of immense quantities of mercury having been given on certain occasions, &c. I well remember to have heard a physician of respectability declare, that in the course of 24 hours a patient of his own had taken an ounce of calomel before his mouth was affected!

I am aware that many may think these remarks strange, or even illiberal. However, as the result of my practice, which I again mention, has been pretty considerable, has been so very different from that of many others; I hope these gentlemen in particular, and the public at large, will in some measure pardon the observations I have made.

I am, Gentlemen,

THOS. CLARK, M.D.

